# The effects of corticotropin-releasing factor on motor learning

**DOI:** 10.1038/s41598-024-66736-0

**Published:** 2024-07-24

**Authors:** Eri Takeuchi, Tomomi Hatanaka, Takatoshi Iijima, Minoru Kimura, Akira Katoh

**Affiliations:** 1 Institute of Innovative Science and Technology, Tokai University, 143 Shimokasuya, Isehara, Kanagawa 259-1193 Japan; 2 Tokai University School of Medicine, 143 Shimokasuya, Isehara, Kanagawa 259-1193 Japan; 3 Course of Pharmacy, Graduated School of Pharmaceutical Sciences, Josai University, 1-1 Keyakidai, Sakado, Saitama 350-0295 Japan; 4 Department of Physiology, Tokai University School of Medicine, 143 Shimokasuya, Isehara, Kanagawa 259-1193 Japan; 5 Present Address: Department of Molecular Therapy, National Institute of Neuroscience, National Center of Neurology and Psychiatry, 4-1-1 Ogawahigashi, Kodaira, Tokyo 187-8502 Japan

**Keywords:** Cerebellum, Motor learning, Corticotropin-releasing factor, Cerebellum, Stress and resilience

## Abstract

Corticotropin-releasing factor (CRF) is mainly secreted from the hypothalamic paraventricular nuclei and plays a crucial role in stress-related responses. Recent studies have reported that CRF is a neuromodulator in the central nervous system. In the cerebellum, CRF is essential for the induction of long-term depression (LTD) at the parallel fiber-Purkinje cell synapses. Given that LTD is thought to be one of the fundamental mechanisms of motor learning, CRF may affect motor learning. However, the role of CRF in motor learning in vivo remains unclear. In this study, we aimed to examine the role of CRF in motor learning. This was achieved through a series of behavioral experiments involving the in vivo administration of CRF and its antagonists. Rats injected with CRF directly into the cerebellum exhibited superior performance on the rotarod test, especially during initial training phases, compared to control subjects. Conversely, rats receiving a CRF receptor antagonist demonstrated reduced endurance on the rotating rod compared to controls. Notably, CRF mRNA expression levels in the cerebellum did not show significant variance between the CRF-injected and control groups. These findings imply a critical role of endogenous CRF in cerebellar motor learning and suggest that exogenous CRF can augment this process. (199 words)

## Introduction

Corticotropin-releasing factor (CRF), a 41-amino acid peptide, plays a crucial role in stress-related responses through the hypothalamic–pituitary–adrenal (HPA) axis^1–3^. In the HPA axis, CRF is secreted from the hypothalamic paraventricular nuclei (PVN) and stimulates cells in the anterior pituitary gland to promote the secretion of adrenocorticotropic hormone (ACTH). ACTH promotes the secretion of glucocorticoids from the adrenal cortex.

CRF has been reported to be involved in stress-related disorders^4,5^. In the central nervous system, CRF is considered a stress-responsive factor and neuromodulator, and its receptors are widely expressed in the brain^6–8^. For example, CRF immunoreactive neurons and fibers were found in the PVN, dorsomedial hypothalamic nucleus, and arcuate nucleus in the rat brain^9^. CRF receptor type 2 (CRFR-2) mRNA is also highly expressed in the hypothalamus^6^. In rodents, CRF and its receptors play roles in anxiety-like behaviors and function, locomotor activity, learning, attention, working memory, spatial memory, and decision-making, independent of the HPA axis^10–17^. CRF is highly expressed in the cortex, bed nucleus of the stria terminalis, central amygdala, anterior cortical amygdaloid nucleus, Barrington’s nucleus, inferior olivary nucleus, and dorsolateral tegmental area in the rat brain^9^. The climbing and mossy fibers in the cerebellum were also CRF immunoreactive^9^. It is well known that the amygdala is a central circuit of fear and anxiety^18^, and the cerebellum has crucial roles in controlling posture, locomotion, and learning^19–23^. CRF receptor type 1 (CRFR-1) mRNA is highly expressed in these brain regions^6^.

A study showed that CRF-overexpressing mice had a significantly smaller cerebellum and whole brain and showed motor dysfunction in the beam test^24^. Mice with downregulated *Crf* mRNA in the inferior olive, the origin of cerebellar climbing fibers (CFs), exhibited motor deficits in the beam and rotarod tests^25^. Mice depleted of CRFR-1 in cerebellar granular cells showed accelerated motor learning on eyeblink conditioning but no changes in motor performance or fear- and anxiety-related behaviors^26^. Mice with inferior olivary neuron-specific CRF knockdown (50% reduced signaling) showed impaired motor performance in the rotarod test but normal home-cage locomotion and anxiety-related behaviors^27^. CRF deficiency in the olivo-cerebellar system induces ataxia-like motor abnormalities^25^, suggesting that CRF released from the inferior olivary neurons is important for controlling gait, posture, and motor coordination. In rats and mice, mossy fibers in the cerebellum contain abundant CRF but not their neurons^9^. The inferior olivary nuclei contain CRF in both the neurons and CFs^9,28^. It was reported that CRF is essential for the induction of long-term depression (LTD) at the parallel fiber-Purkinje cell synapses^29^, a form of synaptic plasticity known to be one of the fundamental mechanisms of motor learning^30^. As CRF is necessary for the induction of LTD using brain slices, it may contribute to motor learning in behaving animals, although no previous study has provided direct evidence for the role of CRF in motor learning in vivo.

In this study, we aimed to examine the role of CRF in motor learning and conducted behavioral studies to analyze the effects of CRF and its antagonists. We found that rats injected with CRF directly into the cerebellum exhibited superior performance on the rotarod test, especially during the initial training phases, compared to control rats. Conversely, rats receiving a CRF receptor antagonist showed less endurance on the rotating rod compared to controls.

## Results

### Effects of CRF infusion in the cerebellum on motor learning

**Figure 1:**
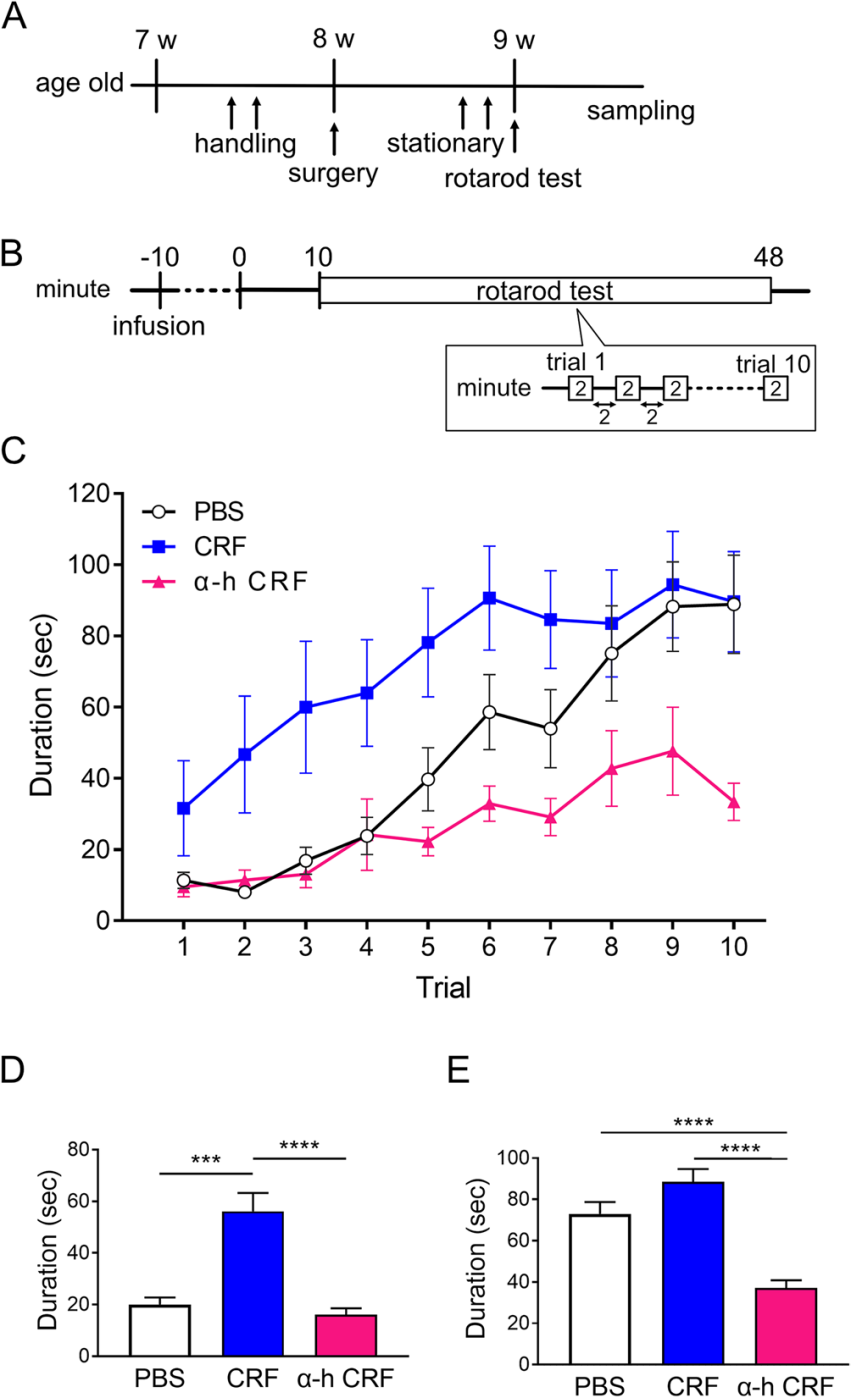
The effect of CRF infusion into the cerebellum on motor learning. (**A**) Experimental schedule. (**B**) Time course of rotarod test. (**C**) Rotarod test scores after infusion of PBS (white, circles, n = 8), CRF (blue, squares, n = 8), or α-helical CRF_9-41_ (described as α-h CRF, magenta, triangles, n = 9) into the cerebellum. (**D**) The average walking duration during trials from 1 to 5. (**E**) The average walking duration during trials from 6 to 10. ***, *p* < 0.001, ****, *p* < 0.0001, by Kruskal–Wallis test. Error bars indicate the standard error. CRF, Corticotropin-releasing factor; PBS, Phosphate-buffered saline; α-h CRF, α-Helical CRF_9-41_.

To examine the effects of CRF infusion in the cerebellum on motor learning, we performed the rotarod test 1 week after implanting the guide cannula. Each rotarod test started 10 min after the CRF infusion was administered. Figure 1 delineates the rotarod test scores following the infusion of either phosphate-buffered saline (PBS) as a control, CRF, or α-helical CRF_9-41_ (α-h CRF), a nonselective CRF receptor antagonist, into the lobule V–VI of the cerebellar vermis. We observed a progressive increase in walking duration across all trials in each group, indicative of motor learning. The CRF-injected rats exhibited higher performance scores on the rotating rod than those exhibited by rats injected with PBS (treatment: F (2, 22) = 14.15, *p* = 0.0001, two-way repeated measure analysis of variance [ANOVA], Supplementary Table 1, Fig. 1C). In the initial half of the trials, the CRF-injected rats exhibited significantly prolonged walking durations on the rotating rod compared to the control group (PBS-treated: 20.0 ± 2.8 s, CRF-treated: 56.1 ± 7.2 s, α-h CRF-treated: 16.1 ± 2.5 s, *p* < 0.0001, Kruskal–Wallis test, Supplementary Table 2, Fig. 1D). In the last half of the trials, the difference between the PBS- and CRF-treated groups gradually reduced (PBS-treated: 73.0 ± 5.7 s, CRF-treated: 88.6 ± 6.2 s, α-h CRF-treated: 37.2 ± 3.7 s, *p* < 0.0001, Kruskal–Wallis test, Supplementary Table 2, Fig. 1E). The control group exhibited a progressive increment in walking duration across successive trials. Conversely, the increase in walking duration for the α-h CRF group was marginally less pronounced compared to that of other groups (Fig. 1C). In the last half of the trials, the average duration in the α-h CRF group was significantly shorter than that in the other two groups (PBS vs α-h CRF: *p* < 0.0001, CRF vs α-h CRF: *p* < 0.0001, Supplementary Table 2, Fig. 1E). These results suggest that one-shot injection of CRF into the cerebellum promotes initial motor learning and that α-h CRF hinders the acquisition of motor learning.

### CRF mRNA expression after infusion of CRF in the cerebellum

**Figure 2:**
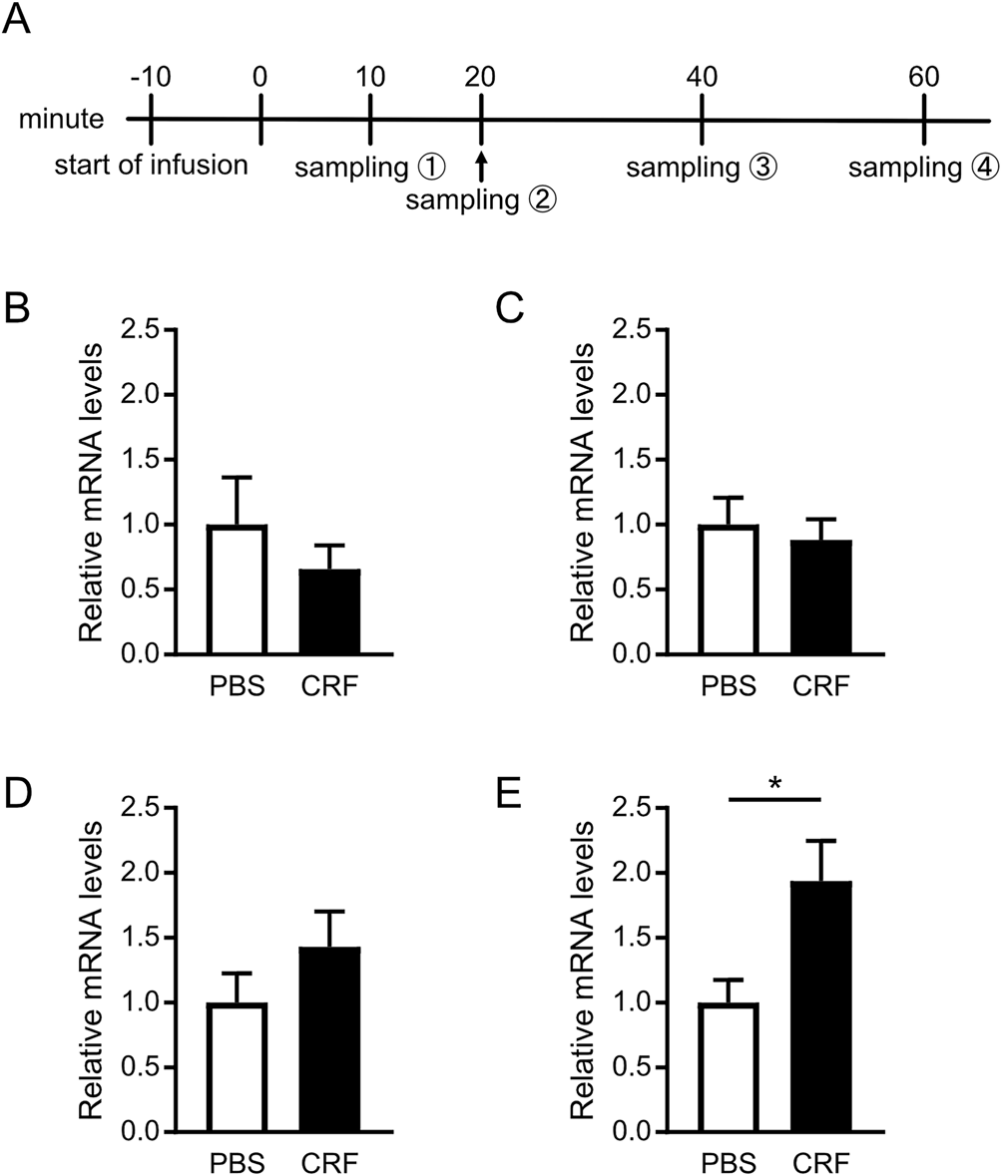
The effect of CRF infusion into the cerebellum on endogenous CRF expression. Each panel shows the relative expression levels of CRF mRNA in the cerebellum 10 min (PBS: n = 6, CRF: n = 6) (**A**), 20 min (PBS: n = 6, CRF: n = 8) (**B**), 40 min (PBS: n = 7, CRF: n = 7) (**C**), and 60 min (PBS: n = 6, CRF: n = 7) (**D**) after infusion of CRF. *, *p* < 0.05 by *t*-test. CRF, Corticotropin-releasing factor; PBS, Phosphate-buffered saline.

To examine how CRF mRNA expression in the cerebellar cortex is modulated by CRF infusion, we compared the relative expression levels of CRF mRNA in the cerebellum at 10, 20, 40, and 60 min after CRF infusion (Fig. 2). Quantitative polymerase chain reaction (PCR) analysis revealed that the levels of CRF mRNA did not differ between CRF- and PBS-injected rats until 40 min. The CRF mRNA levels in CRF-injected rats were higher than those in PBS-injected rats 60 min after injection (t (10) = 0.8525, *p* = 0.4139, Fig. 2B; t (12) = 0.4683, *p* = 0.6480, Fig. 2C; t (12) = 1.22, *p* = 0.2461, Fig. 2D; t (11) = 2.501, *p* = 0.0295, Fig. 2E). These results indicated that the expression of endogenous CRF did not change when animals performed the rotarod test. Exogenous CRF infusion into the cerebellum also clearly induced c-Fos expression, an indicator of neuronal activity (Supplementary Fig. 1). These results suggest that the effect of CRF infusion into the cerebellum on the score of rotarod tests was caused by the exogenous CRF injected (Fig. 1). The delayed increase in CRF mRNA levels observed 60 min after CRF injection may have been due to the reaction time of exogenous CRF to induce the expression of endogenous CRF or stress induced by the high performance of CRF-injected rats.

### Plasma corticosterone (CORT) levels after CRF infusion

**Figure 3:**
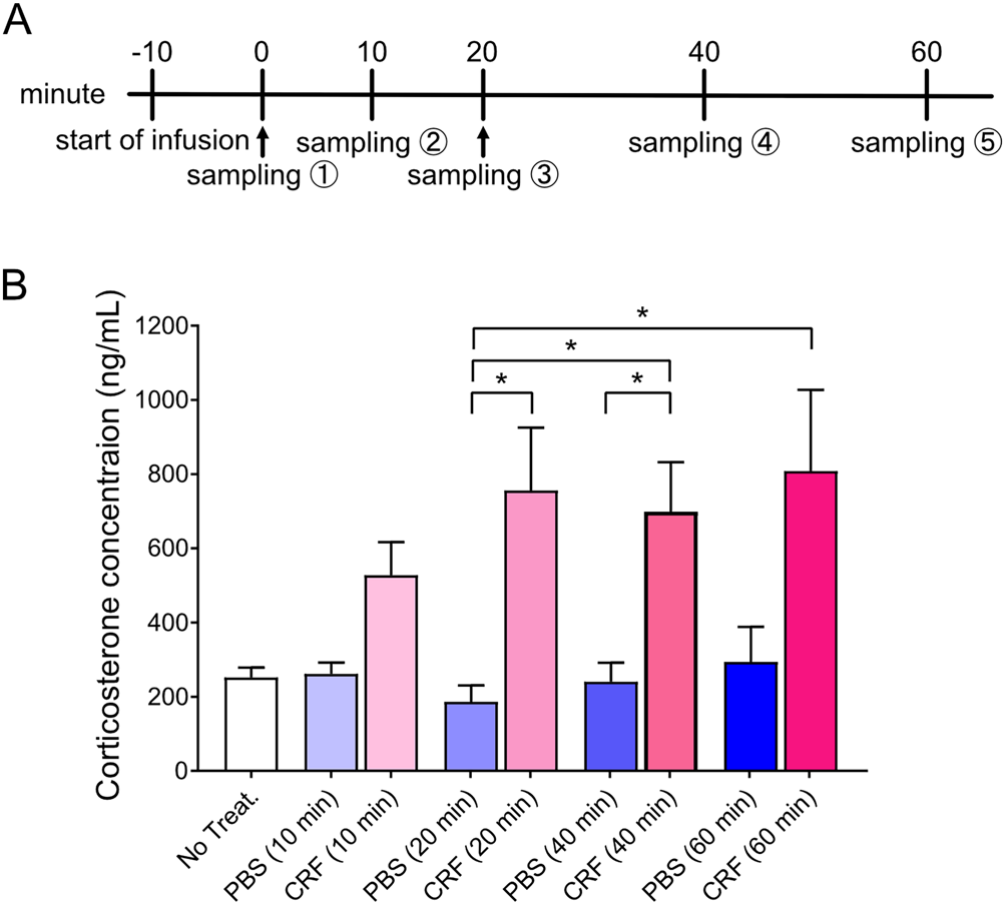
Plasma corticosterone concentration after infusion of CRF. (**A**) Time course of blood sampling. Blood was collected at 0 (no treatment, sampling ①), 10 (sampling ②), 20 (sampling ③), 40 (sampling ④), and 60 (sampling ⑤) after CRF injection. (B) Corticosterone concentrations at different times. “No treat.” indicates that the rats were not injected with any solution after implanting a guide cannula. The mean values for the CRF group were calculated from data obtained from eight animals, while the averages for the remaining two groups were derived from nine animals. *, *p* < 0.05 by *post-hoc* Dunn’s test.

The CORT levels of PBS-injected rats showed no significant differences compared to that of the untreated rats 10, 20, 40, and 60 min after infusion; however, the CORT levels of CRF-injected rats at 20 min and 40 min were higher than that of those treated with PBS for 20 min and 40 min respectively. (*p* < 0.0001 with Kruskal–Wallis test; Not treated: 251.7 ± 27.0 ng/mL, PBS-treated [10 min]: 261.7 ± 30.6 ng/mL, CRF-treated [10 min]: 527.4 ± 89.3 ng/mL, PBS-treated [20 min]: 186.5 ± 43.9 ng/mL, CRF-treated [20 min]: 755.6 ± 169.5 ng/mL, PBS-treated [40 min]: 240.7 ± 50.9 ng/mL, CRF-treated [40 min]: 698.7 ± 133.1 ng/mL, PBS-treated [60 min]: 294.0 ± 94.4 ng/mL, CRF-treated [60 min]: 808.4 ± 218.5 ng/mL, PBS-treated [20 min] vs CRF-treated [20 min]: *p* = 0.0124, PBS-treated [40 min] vs CRF-treated [40 min]: *p* = 0.0471, Fig. 3, Supplementary Table 3). This result suggests that CRF infused into the cerebellum promotes the secretion of CORT, even though the insertion of the injector or infusion itself did not stress the rat.

**Figure 4:**
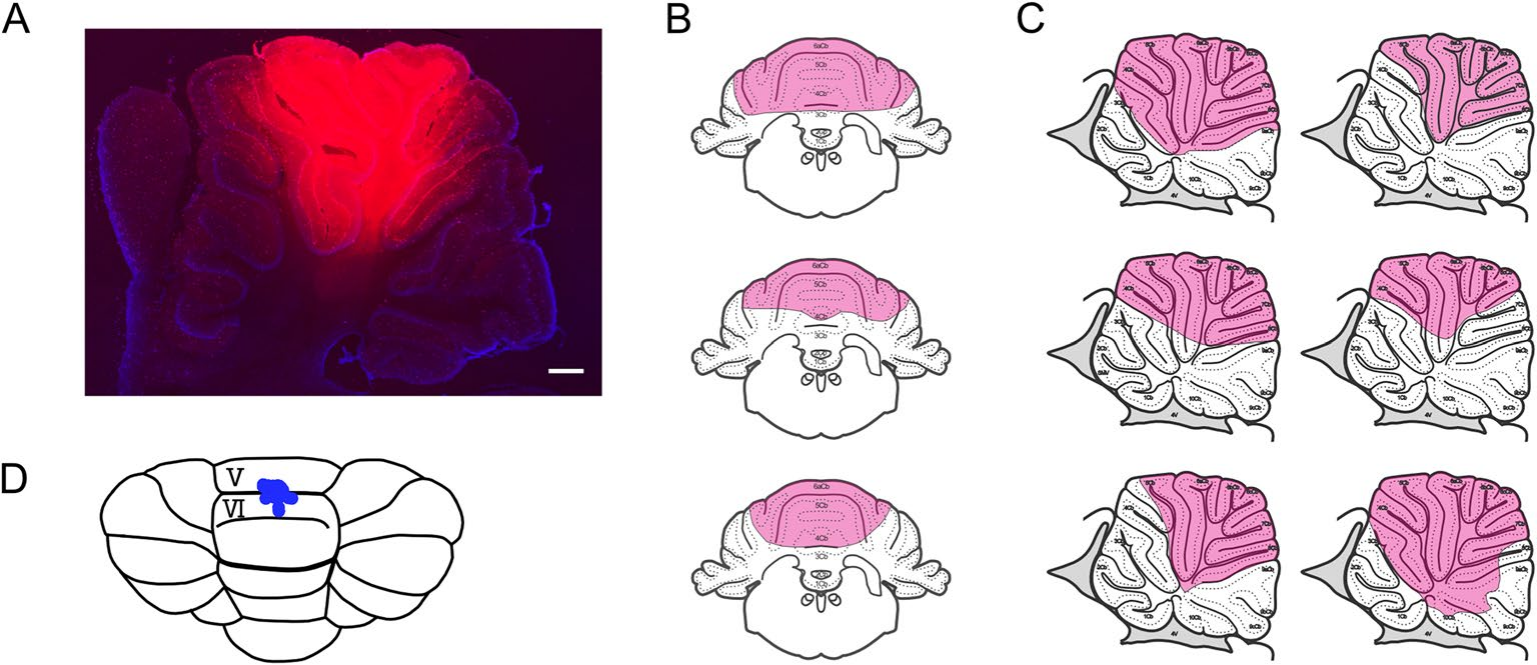
Injection site and diffusion of injected dye in the cerebellum. (**A**) The sagittal section of the cerebellar vermis with dye injection. The red dye is Texas Red, and the blue dye is DAPI. Scale bar indicates 300 μm. (**B**) and (**C**) Schematics showing the diffusion of Texas Red in the coronal and sagittal sections, respectively^48^. (**D**) Schematic showing the injection site.

To confirm the distribution of the drugs, the dye was injected into the cerebellar vermis in the same manner as that for drug solutions (coronal: n = 3, sagittal: n = 6, Fig. 4). In the sagittal section, the dye was distributed from lobules III to IX (Fig. 4A,C) and especially deeper from lobules V to VII than in other areas. In the coronal section, the dye was distributed in the vermis (Fig. 4B). The injection sites were lobules V or VI (Fig. 4D).

## Discussion

In this study, we focused on the role of CRF in motor learning in naïve rats. We showed that the rotarod score of rats injected with CRF into the cerebellum improved compared with that of rats injected with PBS, especially in the first half of the trials. Rats injected with the CRF receptor antagonist exhibited lower rotarod test scores than those injected with PBS.

Some studies have suggested that CRF is involved in motor performance. CRF deficiency in the inferior olive induces an ataxic-like motor deficit^25^. Moreover, CRF in the inferior olive plays a role in motor performance in rotarod tests^27^. The inferior olive-CRF knockdown mice showed impaired motor performance in the rotarod test, but home-cage locomotion was not impaired^27^. We found a significant improvement in the rotarod test scores in rats injected with CRF into the cerebellum compared to those injected with PBS, especially during the early phase of trials. In contrast, the rats injected with α-h CRF, a CRF receptor antagonist, exhibited impaired motor learning in the rotarod test. These results suggest that CRF plays an important role in motor performance and the acquisition of motor learning in the rotarod test. CRF is required for the induction of LTD^29^, which is a fundamental mechanism of motor learning^31^. The application of CRF increases the Purkinje cell firing rate in a concentration-dependent manner^32^. Moreover, CRF induces a simple spike-firing rate in a dose-dependent manner under the activity of gamma aminobutyric acid type A receptor^33^. Although the neural mechanism by which CRF affects motor learning acquisition in the rotarod test remains to be elucidated, the present study showed that CRF injection into the cerebellum contributes to motor learning.

It has been unclear whether changes in the endogenous expression level of CRF mRNA affect motor coordination driven by the cerebellum. We examined changes in the endogenous expression of CRF mRNA after CRF injection in the cerebellum in this study. CRF mRNA expression did not change in either the CRF-injected or control groups up to 40 min after injection (Fig. 2B–D). This result suggests that the improvement in rotarod scores observed in the CRF-injected group was caused purely by exogenous, not endogenous, CRF. In this study, the CRF mRNA levels 60 min after CRF injection significantly increased compared to the control group (Fig. 2E), likely because exogenous CRF stimulated endogenous CRF expression. Another possibility is that their higher performance in the rotarod test after CRF injection might induce stress, resulting in delayed CRF expression. It was reported that CRF mRNA levels in the inferior olivary neurons increased from four- to sevenfold after 48 h of optokinetic stimulation in unanesthetized rabbits and more than tenfold after 144 h of optokinetic stimulation. This optokinetically induced increase in CRF mRNA declined to background levels after 30 h^34^. This suggests that CRF mRNA levels increase in a stimulus time-dependent manner.

Acute stress, such as restraint or hypertonic saline stress, increases CRF mRNA expression in the hypothalamic paraventricular nucleus (PVN) and amygdala^35,36^. In contrast, chronic stress-induced CRF mRNA expression in the PVN increases, decreases, or remains unchanged, depending on the stress stimulation^37–39^. Moderate stress may cause a transient increase in brain CRF levels. Increased expression of *c-fos* mRNA in the limbic structure, hypothalamus, cerebellum, and several brainstem nuclei induced by acute stress, restraint immobilization stress, or foot shock has also been reported^33^. The expression of *c-fos* mRNA is also induced by intracerebroventricular CRF administration in rats^40^. Given that CRF functions as a neuromodulator and is involved in anxiety-related behavior^17,41^, endogenous CRF induced under stress during the rotarod test likely activates neural activity in the cerebellum, resulting in motor learning acquisition. In this study, we demonstrated that exogenous CRF infusion into the cerebellum positively affected motor learning in the rotarod test. In fact, we found that injected CRF induced c-Fos expression in the cerebellar cortex (Supplementary Fig. 1). These findings suggest that a transient increase in cerebellar CRF may additionally affect neural activity to accelerate motor learning.

We found a significant increase in CORT levels in plasma after CRF administration (Fig. 3), although the motor coordination of CRF-treated rats was better than that of controls. We hypothesize that a short-term increase in CORT concentration did not affect motor learning because CORT levels at 20 and 40 min after CRF injection significantly increased compared to those treated with PBS for 20 min and 40 min, respectively (Fig. 3). In contrast, a long-lasting increase in CORT concentrations in the plasma would negatively affect motor learning. Repeated CORT injections have been reported to reduce locomotor activity in mice. C57Bl/6J male mice with repeated 6-day CORT administration revealed significantly shorter latencies before falling than those in controls^42^. Performance alterations on the rotarod displayed by CRF-overexpressing mice^17^ or after CRF antagonist injection in the cerebellar nuclei^43^ corroborate the role of CRF in CORT administration. It was also reported that acute treadmill running increases CORT levels in rodents, CORT levels increase in an intensity-(speed, duration) dependent manner, and regular exercise increases basal CORT levels^44^. The CORT levels in plasma indicates stress, and exogenous CORT administration is a well-established method to mimic stress exposure^45^. Endogenous CRF secreted from neurons in the PVN is known to promote the secretion of CORT from the adrenal cortex through ACTH-secreting cells in the anterior pituitary. Further studies are required to examine the relationship between CRF and CORT as stress factors in the HPA axis and those as neuromodulators in the cerebellum.

## Conclusion

This investigation highlights the crucial role of CRF in cerebellar motor learning. Administration of CRF and its receptor antagonists into the cerebellum enhanced and impaired motor learning in the rotarod test, respectively. Our results underscore the importance of endogenous CRF for cerebellar motor learning and suggest that exogenous CRF can augment this process. The methodologies employed in this study and anticipated future research have significant implications for understanding the interplay between stress and motor learning. This could be instrumental in developing effective training paradigms that incorporate stress considerations for motor skill acquisition.

## Methods

### Animals

Experiments were performed using male naïve Wistar rats aged 9 weeks (weight, 240–306 g; CREA, Japan). All animals were provided food and water ad libitum and maintained in constant conditions (12:12 light/dark cycle, 23 ± 2 °C). All experimental procedures were performed in accordance with the National Institute of Health Guidelines for the Care and Use of Laboratory Animals and were approved by the Institutional Animal Care and Use Committee at Tokai University. All efforts were made to minimize the number of animals used and any discomfort or suffering throughout the experiments in accordance with ARRIVE guidelines^46^.

### Drugs

CRF (C3042, Sigma-Aldrich, Tokyo, Japan) and α-helical CRF_9-41_ (α-h CRF, C2917, Sigma-Aldrich), the nonselective CRF receptor antagonist, were dissolved in PBS. CRF (1 μg/μL), α-h CRF (2.5 μg/μL), or PBS were injected into the cerebellum at a rate of 1 μL/min. It has been reported that CRF intraventricular administration shows dose-dependent locomotor activation at doses between 0.1 and 10 μg^47^. Therefore, we investigated the effect of high concentrations of CRF injection on locomotor behavior, endogenous CRF mRNA expression, c-Fos expression, and CORT secretion.

### Surgical procedures

A guide cannula was implanted into the skull above the cerebellum to inject the drug solution. The animals were anesthetized using isoflurane (3% initial, 1.5–2% maintenance) and placed on a stereotaxic apparatus. To prevent the guide cannula from detaching from the skull, two anchor screws were implanted in the parietal bone. A small hole was created using a drill at the end of the intraparietal bone just above the cerebellar lobule V–VI (Supplemental Fig. 2). The guide cannula (24-gauge, 2 mm long) was implanted into the hole and fixed with dental cement. The guide cannula was filled with a 30-gauge needle to prevent blood congestion. After these procedures, the animals were allowed to rest for 7 days.

### Behavioral tests

The rotarod test was used to evaluate the acquisition of motor learning. Before initiating the test, all rats were handled for 5 min × 3 times/day for 2 days for acclimatization. The rats were habituated to a rotarod apparatus (Shinano, Tokyo, Japan) by being placed on a static rod for 5 min × 3 trials/day for 2 days before the rotarod test. On the day of the rotarod test, the drug solution was injected through a 30-G injector inserted into the implanted cannula without anesthesia. Precisely 10 min after infusion, the rats were placed on a 7 cm-diameter rod rotating at a rate of 20 rpm, and the duration of walking on the rotating rod was measured. The maximum retention time was set to 120 s. Each rat performed the rotarod test in ten trials with intertrial intervals of 120 s.

### Real-time quantitative PCR for assessing CRF mRNA expression

The cerebella were harvested from age-matched rats used in the behavioral tests at 10, 20, 40, and 60 min after CRF or PBS injection. Subsequently, the cerebella were stored at − 80 °C until real-time quantitative PCR (qPCR) analysis. Total RNA was isolated using TRIzol reagent (Thermo Fisher Scientific, Tokyo, Japan) and treated with DNase I (DNase-free, NIPPON GENE, Tokyo, Japan). cDNAs were synthesized using a High-Capacity cDNA Reverse Transcription Kit (Applied Biosystems, Tokyo, Japan). qPCR was performed using StepOne Plus (Applied Biosystems). Custom primers (Table 1) were used with Fast SYBR Green Master Mix (Applied Biosystems). The mRNA levels were normalized to those of *Gapdh* mRNA.

**Table 1:** Primer sequences for qPCR.

Primer	Sequence (5′–3′)	
CRF	Forward	TCACCTTCCACCTTCTGAGG
	Reverse	AAGCGCAACATTTCATTTCC
GAPDH	Forward	AAGGGCTCATGACCACAGTC
	Reverse	GGATGCAGGGATGATGTTCT

CRF, Corticotropin-releasing factor; GAPDH, Glyceraldehyde-3-phosphate dehydrogenase.

### Measurement of plasma CORT concentrations

Blood was collected from age-matched rats used in the behavioral tests. Blood was collected from the hearts under carbon narcosis at 0 (no treatment), 10, and 60 min after CRF injection. Plasma CORT concentration, an indicator of stress, was measured using a Corticosterone ELISA Kit (Assay Pro, MO, USA) according to the manufacturer’s instructions. Plasma samples were obtained from the blood via centrifugation (2000 g, 20 min, 4 °C) and stored at − 80 °C until measurement.

### Histology

To evaluate the extent of diffusion of injected CRF, 10 μL of 0.5% Dextran-conjugated Texas Red (3000 MW, Invitrogen Corporation, Tokyo, Japan) was injected into the cerebellum in the same manner through the implanted cannula in 9 rats, and the rats were then transcardially perfused with saline, followed by 4% paraformaldehyde in PBS. The removed brains were postfixed overnight in the same fixative and stored in 5% and 10% sucrose in 0.1 M Phosphate buffer (PB) for 4 h and then in 20% sucrose in 0.1 M PB for 1 d. The sagittal sections (40 μm) of the cerebellum were prepared with a cryostat (Leica, Tokyo, Japan), collected in 0.1 M PB, mounted on a microscope slide, and covered with cover glass using 1.25 mg/mL DABCO in 10% 0.01 M PBS/glycerol (pH 8.81) with DAPI. The extent of diffusion at the dye injection site was photographed using a digital microscope (KYENCE CORPORATION, Osaka, Japan).

### Statistical analysis

All data were analyzed using GraphPad Prism7 with the Student’s *t*-test, one-way analysis of variance (ANOVA), two-way repeated measures ANOVA, and Tukey’s *post-hoc* test. The Shapiro–Wilk test was used to check the normal distribution of the data, and the Bartlett test was used to check the equality of the error variances. If the data were inconsistent with normal distribution, analysis was performed using the nonparametric Kruskal–Wallis test, followed by Dunn’s test. The results are presented as means ± standard error of the mean. Statistical significance was set at *p* < 0.05.

### Supplementary Information

Supplementary Information.

## Data Availability

The datasets analyzed during the current study are available from the corresponding author on reasonable request.
