# Supplementary material for: The effects of corticotropin-releasing factor on motor learning
Source: Sci Rep. 2024 Jul 24;14:17056. doi: 10.1038/s41598-024-66736-0 (PMC11269602; doi:10.1038/s41598-024-66736-0)
Supplement: Supplementary file 1 — Supplementary Information. [file 41598_2024_66736_MOESM1_ESM.pdf]

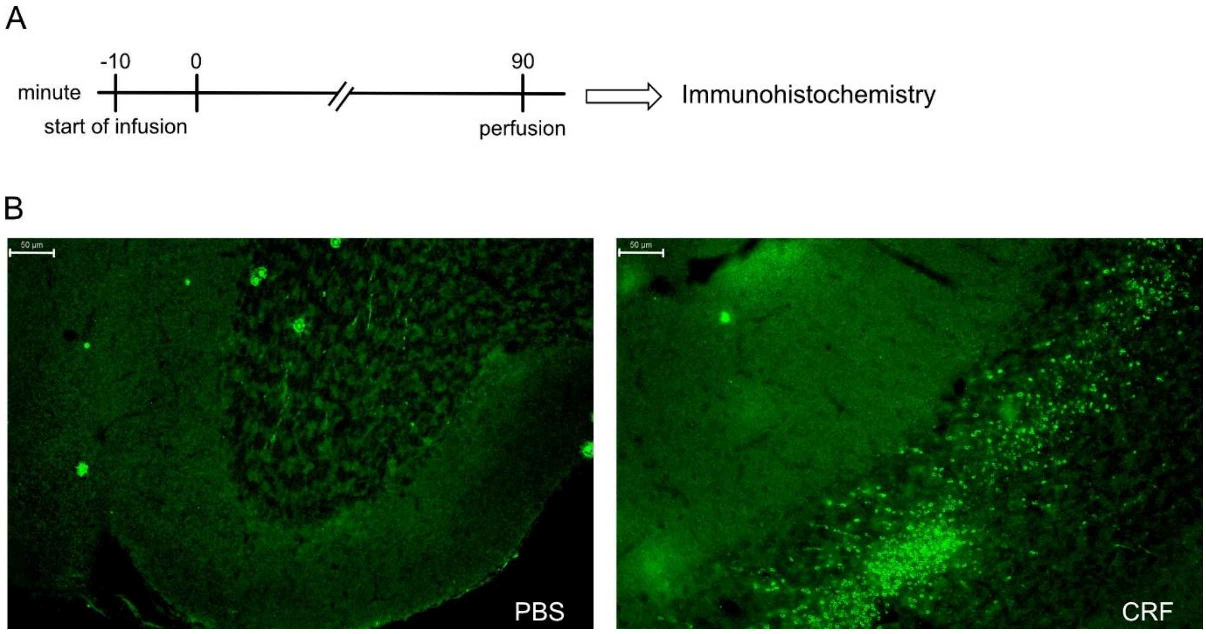

Supplementary Figure 1. c-Fos expression in the cerebellum 90 min after CRF injection.

(A) Time course of brain sampling. The rats were transcardially perfused with saline, followed by 4% paraformaldehyde in PBS 90 min after CRF injection. The coronal sections (14-µm) were prepared with a cryostat. (B) c-Fos immunoreactivity (anti-c-Fos antibody, SAB5700610, Sigma-Aldorich, St. Louis) in the cerebellum of a PBS-injected rat (left panel) and a CRF-injected rat (right panel). The CRF-injected rat showed high immunoreactivity to c-Fos, while the PBS-injected rat did not. Scale bars: 50 µm

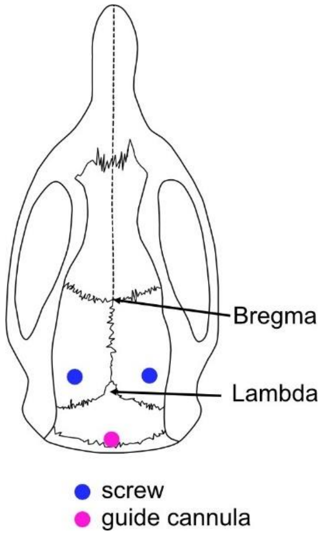

Supplementary Figure 2. Anchor screws and guide cannula localization.

The guide cannula was implanted at the end of the intraparietal bone, just above cerebellar lobule VI. To prevent the guide cannula from detaching from the skull, two anchor screws were implanted in the parietal bone.

Statistical results in Figure 1C.

Supplementary Table 1.

2-way repeated measures ANOVA

	F (DFn, DFd)	P value
Interaction effects	F (18, 198) = 1.445	P =0.1143
Trial	F (9, 198) = 14.98	P <0.0001
Treatment	F (2, 22) = 14.15	P =0.0001

Tukey's multiple comparison test

trial		P value
1	PBS vs CRF	0.3975
	PBS vs α-h CRF	0.9921
	CRF vs α-h CRF	0.3141
2	PBS vs CRF	0.0371
	PBS vs α-h CRF	0.9739
	CRF vs α-h CRF	0.0538
3	PBS vs CRF	0.0169
	PBS vs α-h CRF	0.9666
	CRF vs α-h CRF	0.0063
4	PBS vs CRF	0.0288
	PBS vs α-h CRF	0.9997
	CRF vs α-h CRF	0.025
5	PBS vs CRF	0.0387
	PBS vs α-h CRF	0.4804
	CRF vs α-h CRF	0.0008
6	PBS vs CRF	0.1024
	PBS vs α-h CRF	0.2081
	CRF vs α-h CRF	0.0005
7	PBS vs CRF	0.1237
	PBS vs α-h CRF	0.2301
	CRF vs α-h CRF	0.0009
8	PBS vs CRF	0.8531
	PBS vs α-h CRF	0.0853
	CRF vs α-h CRF	0.0211
9	PBS vs CRF	0.9185
	PBS vs α-h CRF	0.0216
	CRF vs α-h CRF	0.0065
10	PBS vs CRF	0.9987
	PBS vs α-h CRF	0.0009
	CRF vs α-h CRF	0.0008

Supplementary Table 2. Statistical results in Figure 1D, E.

first half

Kruskal-Wallis test

P value	<0.0001
number of groups	3
Kruskal-Wallis statistic	24.89

Dunn test

	P value	
PBS vs CRF	0.0007	***
PBS vs α-h CRF	0.9276	ns
CRF vs α-h CRF	<0.0001	****

last half

Kruskal-Wallis test

P value	<0.0001
number of groups	3
Kruskal-Wallis statistic	35.92

Dunn test

	P value	
PBS vs CRF	0.657	ns
PBS vs α-h CRF	<0.0001	****
CRF vs α-h CRF	<0.0001	****

Supplementary Table 3. Statistical results in Figure 3.

Kruskal-Wallis test

P value	<0.0001
number of groups	9
Kruskal-Wallis statistic	38.01

Dunn test

	P value	
No Treat. vs PBS (10 min)	>0.9999	ns
No Treat. vs PBS (20 min)	>0.9999	ns
No Treat. vs PBS (40 min)	>0.9999	ns
No Treat. vs PBS (60 min)	>0.9999	ns
No Treat. vs CRF (10 min)	>0.9999	ns
No Treat. vs CRF (20 min)	0.2378	ns
No Treat. vs CRF (40 min)	0.206	ns
No Treat. vs CRF (60 min)	0.2553	ns
PBS (10 min) vs PBS (20 min)	>0.9999	ns
PBS (10 min) vs PBS (40 min)	>0.9999	ns
PBS (10 min) vs PBS (60 min)	>0.9999	ns
PBS (10 min) vs CRF (10 min)	>0.9999	ns
PBS (10 min) vs CRF (20 min)	0.356	ns
PBS (10 min) vs CRF (40 min)	0.3102	ns
PBS (10 min) vs CRF (60 min)	0.3811	ns
PBS (20 min) vs PBS (40 min)	>0.9999	ns
PBS (20 min) vs PBS (60 min)	>0.9999	ns
PBS (20 min) vs CRF (10 min)	0.079	ns
PBS (20 min) vs CRF (20 min)	0.0124	*
PBS (20 min) vs CRF (40 min)	0.0104	*
PBS (20 min) vs CRF (60 min)	0.0136	*
PBS (40 min) vs PBS (60 min)	>0.9999	ns
PBS (40 min) vs CRF (10 min)	0.3019	ns
PBS (40 min) vs CRF (20 min)	0.0554	ns
PBS (40 min) vs CRF (40 min)	0.0471	*
PBS (40 min) vs CRF (60 min)	0.0601	ns
PBS (60 min) vs CRF (10 min)	0.5944	ns
PBS (60 min) vs CRF (20 min)	0.1286	ns
PBS (60 min) vs CRF (40 min)	0.1109	ns
PBS (60 min) vs CRF (60 min)	0.1384	ns
CRF (10 min) vs CRF (20 min)	>0.9999	ns
CRF (10 min) vs CRF (40 min)	>0.9999	ns
CRF (10 min) vs CRF (60 min)	>0.9999	ns
CRF (20 min) vs CRF (40 min)	>0.9999	ns
CRF (20 min) vs CRF (60 min)	>0.9999	ns
CRF (40 min) vs CRF (60 min)	>0.9999	ns
